# Dimensions of psychomotor performance: past and future

**DOI:** 10.3389/fpsyg.2026.1717557

**Published:** 2026-04-24

**Authors:** Klaus Bös, Heinz Mechling

**Affiliations:** 1 Institute of Sports and Sports Science, Karlsruhe Institute of Technology (KIT), Karlsruhe, Germany; 2 Institute for Gerontology, German Sport University Cologne (DSHS), Cologne, Germany

**Keywords:** ability approach, application orientation, communality analysis, dimensional analysis, psychomotor performance

## Abstract

Motor performance has been examined from various scientific perspectives and with different methodological approaches. In basic research, the main goal is to identify underlying mechanisms that determine performance. This is typically achieved through the analysis of metabolic parameters and, with regard to the central nervous system, neurophysiological measures. When the concept of “motor performance” is expanded to “psychomotor performance,” an essential dimension of human behavior comes into focus, namely intentionality. Observable behavior is grounded in metabolic and neurophysiological processes and can be described using measurable parameters. However, explaining behavior in complex situations such as demanding tasks, elevated stress, or dynamic social contexts requires more than the analysis of motor processes alone. Rather, action performance needs to be examined, which integrates purposeful and context-sensitive behavior. Restricting explanations to isolated motor parameters raises questions about their adequacy and practical relevance, particularly when interventions are to be derived from them. As a practice-oriented alternative, this article adopts the concept of ability as its central framework. Psychomotor abilities are understood within an action-oriented and biocultural context. The approach is explored from historical, systematic, conceptual, and empirical perspectives. A theoretically grounded and ability-based model serves as the foundation for subsequent empirical studies that investigate the dimensional structure of the constructs used to explain performance and examines their specific contribution to psychomotor performance. In addition to motor-related factors, cognitive, emotional, and social influences, including parental and school contexts, are considered. Validated behavioral testing instruments and statistical procedures, particularly factor and commonality analyses, are applied to identify distinct dimensions and to determine their explanatory value. The dimensional analysis has three main objectives. First, it seeks to identify constructs as distinct and one-dimensional units. Second, it examines the extent to which these units explain variance in complex physical activity performance. Third, it aims to use empirically validated dimensions as a basis for developing targeted intervention strategies. To achieve these aims, a conceptually coherent and empirically testable structural model was developed, addressing the areas of motor, cognitive, emotional, and social development. The data for the primary study carried out in 1983 were derived from a large sample comprising 342 male 10 year old students, 47 indices, and more than 2000 individual measures, and were subsequently analyzed using statistical procedures. The results allowed us to identify clearly defined variable domains, and to determine their respective contributions to explaining complex psychomotor performance. The findings provided the foundation for numerous subsequent empirical studies between 1991 and 2023, which further refined and extended the model over the following decades. From the beginning, the overarching goal was to improve our understanding of psychomotor performance and to derive practical implications. This orientation towards application is particularly evident in fields related to movement, especially health and sport.

## 1 Historical background to dimensions

Even early humans possessed the ability to recognize similarities and differences among objects, plants, animals, and people. Through processes of sorting, grouping, and classifying, they began to identify fundamental dimensions such as length, width, and height that were essential for navigating daily life. This systematic search for classification systems and behavioral dimensions is as old as the study of behavior itself and the quest to understand human performance. Ancient Greek philosophers Plato, Aristotle, and Heraclitus, among others were already deeply engaged in defining categories, classifications, and dimensions. In modern statistical terms, these efforts can be seen as attempts to identify independent descriptive variables that capture the essence of a characteristic or domain as adequately and “sparingly” as possible, thereby reducing informational complexity (Crowson, 1970; Mayr, 1969).

The exploration of factors that describe, explain, and predict human performance spans numerous areas of society such as politics, economics, and culture, encompassing music, literature, theatre, dance, and sport. Wang & Wang show that “performance research” extends beyond movement and sport, encompassing the highly complex field of music (Wang and Yang, 2024). Scientific approaches to this search have long been rooted in both philosophy with its predominantly qualitative, methodological focus or the social and behavioral sciences, which employ both qualitative and quantitative methods. Studies of individual performance are often conducted using hybrid methodological frameworks, particularly within diverse cultural contexts and fields such as exercise and sports sciences. This implies that different types of content and/or methods from different fields must be integrated to establish a valid explanatory approach. The overarching goal in identifying and applying psychomotor dimensions is to use certain characteristics (psychomotor abilities) of individuals in relation to their performance (conditions and causes), inter-individual differences (through classification within a reference framework) and as a basis for predicting behavior (Mechling, 1987).

To the best of our knowledge, several differential approaches exist, each based on distinct theoretical and foundational scientific premises. However, none of these approaches are based on complex behavior, and most lack comprehensive analysis or extensive empirical validation. This paper presents, in detail, the development of a model designed to explain psychomotor performance, including the operationalization of its system components, as well as its empirical implementation and statistical validation.

## 2 Theoretical and methodological considerations for a model to explain psychomotor performance

### 2.1 Theoretical considerations

Particularly in the behavioral and social sciences, it is important to determine whether the intended research is application-oriented or fundamental when selecting theoretical approaches or their corresponding conceptual models. *Application-oriented* refers to people acting in specific situations. Theories and models for basic research have a different character and different objectives than those for the behavioral research and the claim of “action research” considered here. In both cases, however, the selection and definition of theory-appropriate units of analysis as possible variables of influence is important. In basic science, the orientation is towards existing, empirically confirmed, in most cases deterministic-causal theories, inseparably based on experimental methods. When applied to human physical performance, this involves analyzing physical, physiological, and biomechanical parameters in the areas of endurance, strength, anatomy, sensorimotor control and regulation, collectively referred to as coordination (Golle et al., 2022; Mechling, 1998; Mechling, 2003), and psychological parameters related to cognition and emotion. The aim is to identify physical process-related mechanisms extending to the molecular, atomic level. In order to explain and predict complex behavior on the basis of deterministic-causal models, it is necessary to assume that all influencing factors are known and can be operationalized; a condition that is rarely met in practice.

Over time, several theory based approaches from different scientific fields such as psychology, motor control, neuroscience and sport psychology have been analyzed with regard to their contribution to explaining complex behavior or goal-directed control. This research, using several single physiological parameters has led to the realization that the interaction of isolated variables does not necessarily yield predictable behavioral outcomes in complex systems. Research results in basic science on *emergence* (Amaral et al., 2004; Bäckman and Dixon, 1992; Gigerenzer et al., 1999) and *epigenetics* (Jablonka and Lamb, 2005; Miller, 2010) point to possible causes and processes that need to be taken into account. These results will not be discussed in detail in this paper, but their broad existence supports the notion of higher levels of system organization. For the phase transitions that occur in emergent and epigenetic processes (interaction of neurons) there is, or appears to be, no material-measurable substrate (Miller, 2010). These findings make it necessary to accept uncertanties, as pointed out in “biocultural co-constructivism,” which can be seen as a logical step forward from “interactionism via constructivism” (Baltes et al., 2006, 3–39). Even the approach of a “Grand Unified Theory of Sports Performance—GUT” (Glazier, 2015, 148, see below)—as a high aspiration in sport science—must contend with these uncertainties due to the sheer complexity and multitude of interacting influences involved. Even the foundational method of experimental design, rooted in the natural sciences, is called into question when applied to psychological and psychomotor research (Kuhbandner and Mayrhofer, 2025; Lau and Baumeister, 2025). In light of these developments, we concluded that the ability concept, which has existed in behavioral science for more than 100 years, may provide a promising path forward.

Psychomotor abilities comprise certain independent (purely biological) and certain interacting or even overlapping basic (bio-psycho-cultural—selforganizing) elements. Action performance refers to the interplay of motor, cognitive, emotional socio-cultural elements in an acting person. To use an ability in empirical research as influencing factor, evidence of its one-dimensionality must be established through adequate testing and statistical methods. The empirical part of this paper is designed in accordance with this strategy.

### 2.2 Methodological considerations

Acknowledging these uncertainties is more feasible within an action-oriented ability approach. Whether individual parameters or broader ability constructs are applied depends largely on the specific context of application, for example, whether the focus is on health promotion or competitive sport, and whether the objective is to *maximize* physical performance or *optimize* other specific effects. In the case of peak physical performance in sport, the focus is on maximizing performance. By contrast, when the focus is on maintaining physical function in daily life or promoting long-term health, the objective shifts towards optimizing effects, such as minimizing harmful influences or enhancing protective factors, including the preservation of cardiovascular functionality, muscular strength or the regulation of balance.

*Effect optimization* also applies to many areas of everyday life in which physical activity and performance play an important role. One example is the individual design of physical activity in school sport with its sub-aspects of personal *development*, *education*, *training*. Additionally, physical activity contributes to broader social outcomes, including social behavior and integration within school settings as well as in community-based, recreational, and popular sports. From a health and optimization perspective, environmental design also plays an important role. Incentives that encourage movement can have an activating effect; in the case of physical limitations and disabilities, forms of environmental design that ensure and support movement are helpful.

In the broader contexts described, the focus is not solely on observable behavior in isolation, but also on the subjective experience accompanying it. “Any form of performance is inherently tied to specific tasks and their associated demands, which may be externally assigned or self-initiated. Whether simple or complex, such activities are shaped by a range of cultural, social, situational environmental and execution-related influencing factors” (Mechling, 2021, pp. 139f). Performance thus occurs within a comprehensive activity context and constitutes a form of human action, culminating in an outcome, a result, a product, or a measurable achievement. The realization of physical performance should therefore be understood as an ability based psycho-physical process (Baltes et al., 2006, pp. 3ff; Hacker, 2005; Wessel, 2015).

## 3 Theoretical and methodological justification of the ability concept

### 3.1 Psychomotor abilities as units of analysis

In an application-oriented approach, theoretically and behaviorally appropriate units of analysis must reflect the complexity described above, or at the very least, be situated within that complexity by clearly delineating the limits of their scope and validity. From an empirical standpoint, these units should be identifiable, isolable, and verifiable, thereby supporting both practical application and implementation. This means that they can be used for interventions in learning and training practice and for their evaluation.

**Figure 1:**
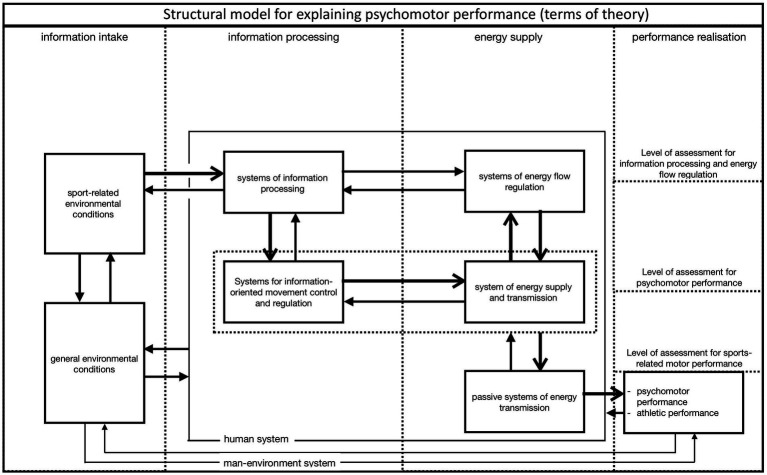
Structural model to explain sports-related movement performance (Adapted from Bös and Mechling, 1983, p. 113; Gundlach, 1968, p. 203).

Basic scientific knowledge must be updated and integrated into the further development of content in order to accurately define complex units of analysis (Figure 1). At the heart of this effort lies a central question: Which application-oriented, theoretically sound, and contextually relevant influencing variables can adequately account for the complexity of human behavior and action?

The aim of this article is to describe, explain, and predict human performance and efficiency from a behavioral and sports-science perspective. A range of scientific disciplines have previously proposed and developed comprehensive theories and methods of analysis.

Over the years, there have been repeated proposals to develop a grand unified theory that accounts for all the factors influencing human physical performance and action (Wolpert et al., 2003). The Universal Theory of Consciousness (Kanai and Fujisawa, 2024) falls within this broader ambition to unify explanatory frameworks across disciplines, as does the Global Work Space Theory of Baars et al. (2021). Glazier’s (2015) “target article” on a “Grand Unified Theory of Sports Performance GUT” is a new proposal on sports performance, the naming clearly echoing the Standard Model of Physics (GUT or even a Theory of Everything (TOE)). Initial commentaries on Glazier’s GUT model (Cardinale, 2017; Rein et al., 2017) generally welcome and acknowledge the thoroughness of its conceptual elaboration. However, they express critical concerns regarding empirical testability. The clear focus on necessary interdisciplinary cooperation as a contribution to the GUT model is assessed more cautiously due to the development of the current state of knowledge and the reality of research. Glazier (p. 139) clearly focuses on sport and high-performance sport. The claim of his model is “to foster interdisciplinary research collaborations; break down the silos that have developed in sports science and restore greater disciplinary balance to the field; promote a more holistic understanding of sport performance across all levels of analysis; increase the explanatory power of applied research work; provide stronger rationale for data collection and variable selection; and direct the development of integrated performance monitoring technologies.” Although the model references potential applications in training practice, it lacks specific criteria, metrics, or testing protocols. However, it is acknowledged that “a significant challenge for applied sports scientists adopting the GUT approach advocated here, however, is the task of measuring and analyzing the effects of different constraints on emergent patterns of coordination and control at both the intra- and inter-individual levels of analysis in real-time during competition” (pp. 139, 150). The GUT approach requires not only interdisciplinary collaboration but also transdisciplinary approaches (Mittelstraß, 2002; Nicolescu, 2002).

As a framework for addressing the complexity of behavior and particularly in relation to learning and training practice, Glazier draws on Newell’s (1986) Constraints Model as a foundational perspective. Newell describes “Functional Movement Behavior” with the sub-areas “*organism*─*task*─*environment*” and the process-related connections for the coordination process with *perception* and *action*. This perspective builds on earlier theoretical foundations, with notable antecedents in sport psychology, activity theory, and action theory.

As early as 1975, Nitsch and Munzert (1997, p. 117) described movement behavior as situation optimization and defined the respective constellation of “*person*─*task*─*environment*” as its fundamental components. In comparison to Newell’s constraint model, it is important to note that Nitsch conceptualizes the “person” from a psychological perspective, as “personality” rather than (in a restricted way) as “organism” (organic) as in the constraint model, which only refers to physical processes. This distinction underscores Nitsch’s emphasis on intentional regulation directed towards specific goals. In addition, fundamental aspects of situation management are taken into account, namely “*demand*” (as an expression of individual *ability*) and “*request*” (as a motivational component), both of which serve as key psychological categories (Nitsch and Munzert, 1997, p. 119). Both Nitsch’s and Newell’s models move beyond strictly biological systems and units, aiming to account for subjective, conscious, and unconscious psychological processes. From an action-theory perspective, “training and exercise science cannot do without a differentiated analysis of the intentional regulation of behavior in sport” (Nitsch and Munzert, 1997, p. 109).

For a realistic, behaviorally grounded, and application-oriented approach, it seems worthwhile to take a step back and consider existing alternative strategies, approaches, theories and models that offer the potential for operationalization and empirical validation. This perspective supports the use of more complex units of analysis than functionally meaningful concepts from fields such as behavioral psychology, including the ability-based approach, and not limiting oneself exclusively to individual biomedical parameters.

### 3.2 Origins of the ability approach

To adopt a realistic, behaviorally grounded, and application-oriented approach, it appears valuable to take a step back and consider existing alternative strategies, approaches, theories and models that offer the potential for operationalization and empirical validation. This perspective supports the use of more complex units of analysis than functionally meaningful concepts from fields such as behavioral psychology, including the ability-based approach, and not limiting oneself exclusively to individual biomedical parameters.

The *ability approach* has two foundational roots. One root originates in general and differential psychology in Germany and the United States around the turn of the 19th and 20th centuries, particularly in the context of intelligence research (Cattell, 1943, 1963; Cattell and Raymond, 1971; Guilford, 1971; James, 1890; Spearman, 1927; Stern, 1900; Wundt, 1905) and in the 1950s with Fleishman’s “human performance research.” Fleishman was instrumental in formalizing the ability approach, asserting that “the development of a given skill or proficiency on a given task is predicated in part on the possessions of relevant basic psychomotor abilities” (Fleishman, 1954; Fleishman et al., 1984, p. 163). His work advanced the search for underlying performance dimensions. Fleishman emphasizes the ability approach to his taxonomy work when he states “the need for human performance taxonomy is linking basic and applied areas” (1984, p. 10). These central demands and questions rest on two essential premises that should not be neglected in psychology and the behavior and movement sciences: that there is neither a situational vacuum nor a person without a developmental and experiential past in action and behavior-oriented research.

The second root of an ability theory is found in Russian occupational psychology from the 1930s to the 1950s, particularly in the works of Leontjew (1982) and Rubinstein (1946–1977, in Russian). Rubinstein stated that “the production of products through the practical and theoretical activity of man presupposes certain abilities the development of these abilities are two mutually interrelated, interdependent, and ultimately inseparable aspects of a unified process of activity and ability” (Rubinstein, 1973, p. 197, 1977, p. 311). A significant influence during this period came from Bernstein’s movement physiology (Bernstein, 1988; Whiting, 1983; Latash and Zatsiorsky, 2015). Drawing on these conceptual psychological and physiological foundations, Gundlach (1968) developed a systems-relational model of physical psychomotor abilities and skills, while Schnabel (1968) proposed a cybernetically oriented model of movement coordination. Both models aimed at explaining the complexity of sports-related motor performance. Due to close academic connections with the Soviet Union, scholars from the former German Democratic Republic gained early access to Russian publications, many of which were translated into German. These works contributed significantly to the dissemination of the ability approach in combination with movement coordination research within German-language sports-science literature (e.g., Hirtz et al., 1985; Schnabel, 1974). In sports science in the Federal Republic of Germany, these ability-based approaches were adopted and further developed in several studies in the 1970s and 1980s (Bös and Mechling, 1983; Eberhardt et al., 2020; Preuß, 2023; Roth, 1982).

The theoretical approach to psychomotor abilities draws upon both the Russian/German sources discussed earlier, as well as US-based research, where “ability concepts” were extensively discussed. Fleishman and Bartlett (1969, p. 350) explained: “The distinction is made between ability and skill, the former being more general to performance across different tasks, and the latter referring to level of proficiency attained on a specific task.” Two further assumptions are associated with the concept of relatively stable psychomotor abilities: first, their relatively high generalizability (i.e., the ability to transfer across various tasks) and second, their transferability (the transfer of a specific ability to different tasks) (Roth, 1999; Schmidt et al., 2019; Schmidt and Wrisberg, 2008). Fleishman (1954) introduced the more complex term “psychomotor abilities” in his research, operationalizing them through psychomotor test procedures, and employing correlation-statistical and factor-analytic methods. As units of analysis, psychomotor abilities have a complex character, which inherently includes certain imprecisions. Consequently, they can only be considered valid units of analysis once their dimensionality has been verified. In American psychology, this approach developed almost in parallel with a theoretical and methodological framework based on the “state–trait” discussion (Bem and Allen, 1974; Cronbach, 1957). This debate revolved around two positions: the differential, correlational-statistical approach focused on test development, and the “situationist” perspective which emphasized experimental methods. The debate continues to this day and also shapes the discussion surrounding the concept of ability. Proposals for addressing these question have been discussed for a long time.

Cronbach (1957) had already criticized the growing divide between “situationists” and “correlationists” advocating for “true federation of both to give answers to the otherwise neglected interaction of organismic and treatment variables.” He concluded that “our job is to invent constructs and to form a network of laws which permits prediction”. These constructs include psychomotor abilities and motor skills which are validated and defined through dimensional analysis. Endler (1973, p. 289) summarized the debate by stating: “Asking whether behavioral variance is due to either situations or to persons is analogous to asking whether air or blood is more essential to life.”

In the present case, the focus is on the trait position in psychomotor abilities and testing procedures. A recent publication by Kuhbandner and Mayrhofer (2025) once again challenges the appropriation of experimental methods in psychological, and by extension, psychomotor research. They emphasize that “law-like behavior in experiments could only occur if truly low-level mechanisms were studied in a truly isolated way (…).” However, unlike in the natural sciences, the mechanisms of the human psyche can only be isolated from one another to a limited extent, as the psyche always responds as a whole system. If such limitations could be overcome, meaningful knowledge might be gained through experimental psychological research. However, the knowledge gained is very limited in terms of its explanatory power for human behavior, as it only aids in understanding a very specific aspect of behavior, namely the mechanistic functioning of isolated low-level mechanisms. To understand motivated behavior in real-life contexts, insight into the non-mechanistic functioning of the higher levels of the human psyche is essential. This type of knowledge, however, cannot be acquired through experimental methods.” These arguments support the strategy to use abilities as higher order research units. The ability concept might be a part of it.

### 3.3 Intelligence research and the ability concept

Intelligence research offers a compelling example of how the dimensionality of cognitive functions and abilities has evolved over decades, resulting in a complex system of traits, functional characteristics, and corresponding tests. Similar approaches can also be applied to the description and measurement of motor behavior. Building on factor analysis, Cattell proposed the foundational dimensions of *fluid* and *crystallized* intelligence, concepts that remain central to intelligence research today. More recent discoveries, informed by advanced neurophysiological methods, have contributed additional dimensions (Goschke, 1996). Wilhelm (2005, 2009) draws upon these distinctions to clarify the diagnostic procedure for intelligence-related terms such as competence, ability and skill.

A central concept that presupposes the differentiation of individual intelligence functions is the concept of ability. Abilities can be described as a unity of knowledge and proficiency. This is to be understood in the same way for the differentiation of motor skills and psychomotor abilities. In analogy to Cattell’s differentiation of intelligence dimensions, *motor skills are crystalline* (knowledge- and proficiency-based) and *psychomotor abilities are fluid* (situational-operational-action based). In sports literature, the term *competence* is often used interchangeably to the ability and skill concept. Therefore, it seems to be of particular importance that a conceptual clarification of ability and skill is appropriate.

A further behavioral and practical perspective is offered by the concept of “agility” (Young et al., 2015). Agility is defined as “rapid whole-body movement in response to an external stimulus.” Operational biomechanically based key elements are the “change of direction [CoD]” and the “change of velocity [CoV],” which result from a complex process of perception and decision-making within the movement task. Agility is thus understood as a construct simplified into two parameters and is therefore not considered an ability. In the terminology of Bös and Mechling (1983, p. 185, p. 191), agility pertains to coordination under time pressure.

### 3.4 Structural model to explain sports-related movement performance

A distinct structural model has been developed to facilitate empirical testing of the ability-based approach outlined above.

The model, inspired by Gundlach (1968, p. 203), posits that individuals act within their respective environments. This systems-behavioral approach presents the latent components and their interrelationships, which contribute to observable movement performance at the manifest level. An attempt is made to represent the identified theoretical constructs and their relationships in the model as realistically as possible. This also applies to the arrow directions and arrow strengths. The superordinate cognitive, emotional (information-oriented) and physiological-neurological (energetic related) mechanisms and processes are located at the top level of the human system. The second level comprises psychomotor abilities, which, based on the first level, can be further classified into information-oriented (coordinative) and energy-related (conditional) psychomotor abilities. The transmission systems of the skeletal system (flexibility) and the anatomical and physical prerequisites (constitution) are pivotal in translating these psychomotor abilities into specific environmental contexts. At the level of realization (performance), a distinction is made between *general psychomotor performance* in motor tests and *sports performance* in competitions.

**Figure 2:**
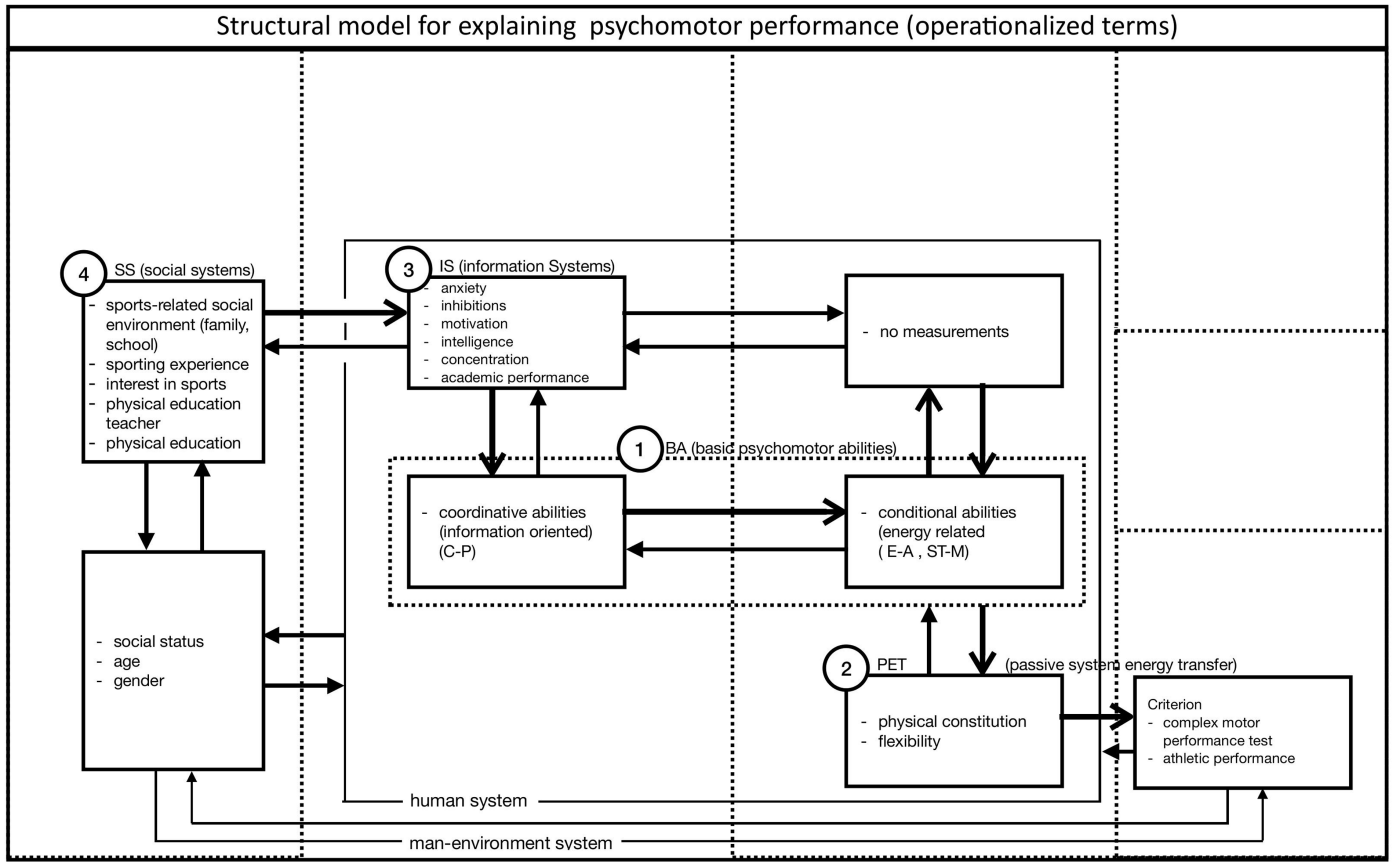
Operationalized structural model to explain sports-related movement performance (Adapted from Bös & Mechling, 1983, p. 119).

Please refer to Chapter 4.2 for a description of how the theoretical concepts are made accessible for operationalization and empirical testing (Figure 2).

## 4 Empirical verifications of the structural model

### 4.1 Questions

In several studies (Chapter 4.3) over a long period of more than 40 years, four main questions have been investigated.

Questions 1 and 2 refer to the test of the model formulated in chapter 3.2 (sample 1): (1) What proportion of the explanation of *complex psychomotor performance* tests is accounted for by uni-dimensional basic psychomotor abilities?; (2) Which components explain complex *psychomotor performance* tests? Question 3 assesses the stability of motor psychomotor abilities over 10 (sample 2) or 20 years (sample 3): (3) How stable are psychomotor abilities over a lifetime? Finally, question 4 further considers the relevance of motor psychomotor abilities for explaining health parameters (sample 4,5 & 6): (4) What is the relevance of psychomotor abilities for health?

### 4.2 Methods

In a first step, the model formulated in 3.4. was operationalized and thus made empirically testable. The model was tested with participants (sample 1) from the baseline study (4.3).

To test the model, the two sets of criteria were used: (1) published complex sports motor tests to assess general motor performance, and (2) performance in the Federal Youth Games, (“Bundesjugendspiele”) which are held in Germany as a school competition.

The tests consisted of 23 tasks such as simple and complex runs, push-ups, jump and reach, etc. The 50 m run, long jump, and ball throw were assessed at Federal Youth Games.

Communality analyses were calculated as a statistical method for model testing (Cooley and Lohnes, 1976). Communality analysis quantifies the variance in the criterion variable explained by a set of predictors.

In the present model test, four levels were calculated (Bös and Mechling, 1983, p 236ff; Figures 4 and 5).

**Figure 3:**
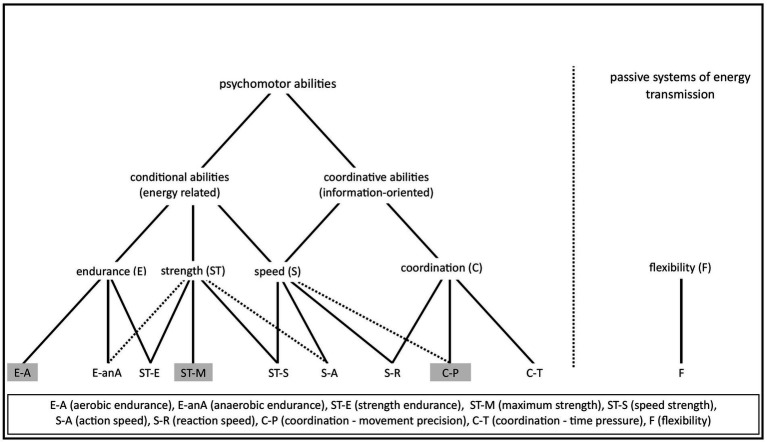
Differentiation of psychomotor abilities (adapted from Bös, 1987, p. 94).

**Figure 4:**
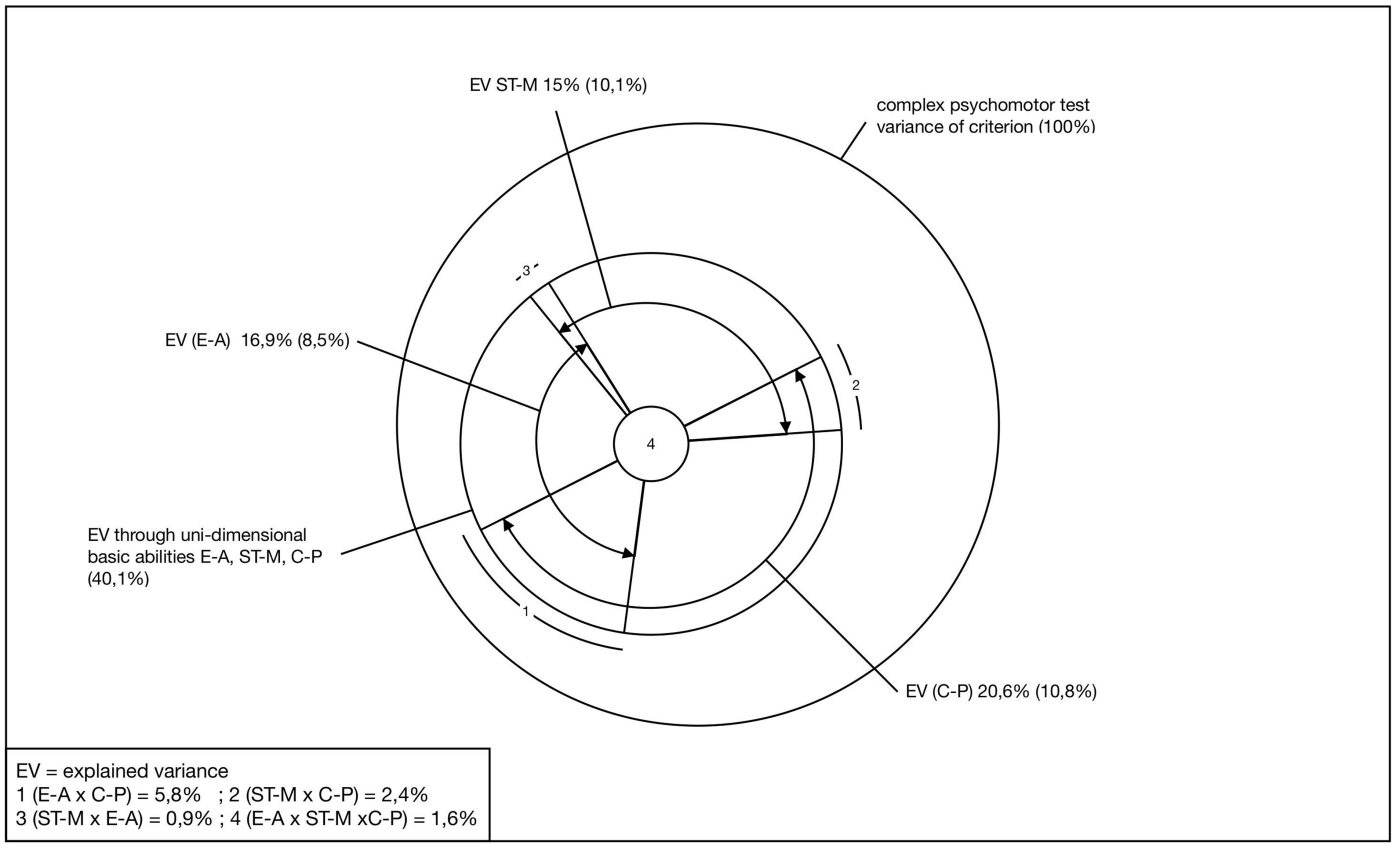
Stage 1 of the model test (Adapted from Bös & Mechling, 1983, p. 241).

**Figure 5:**
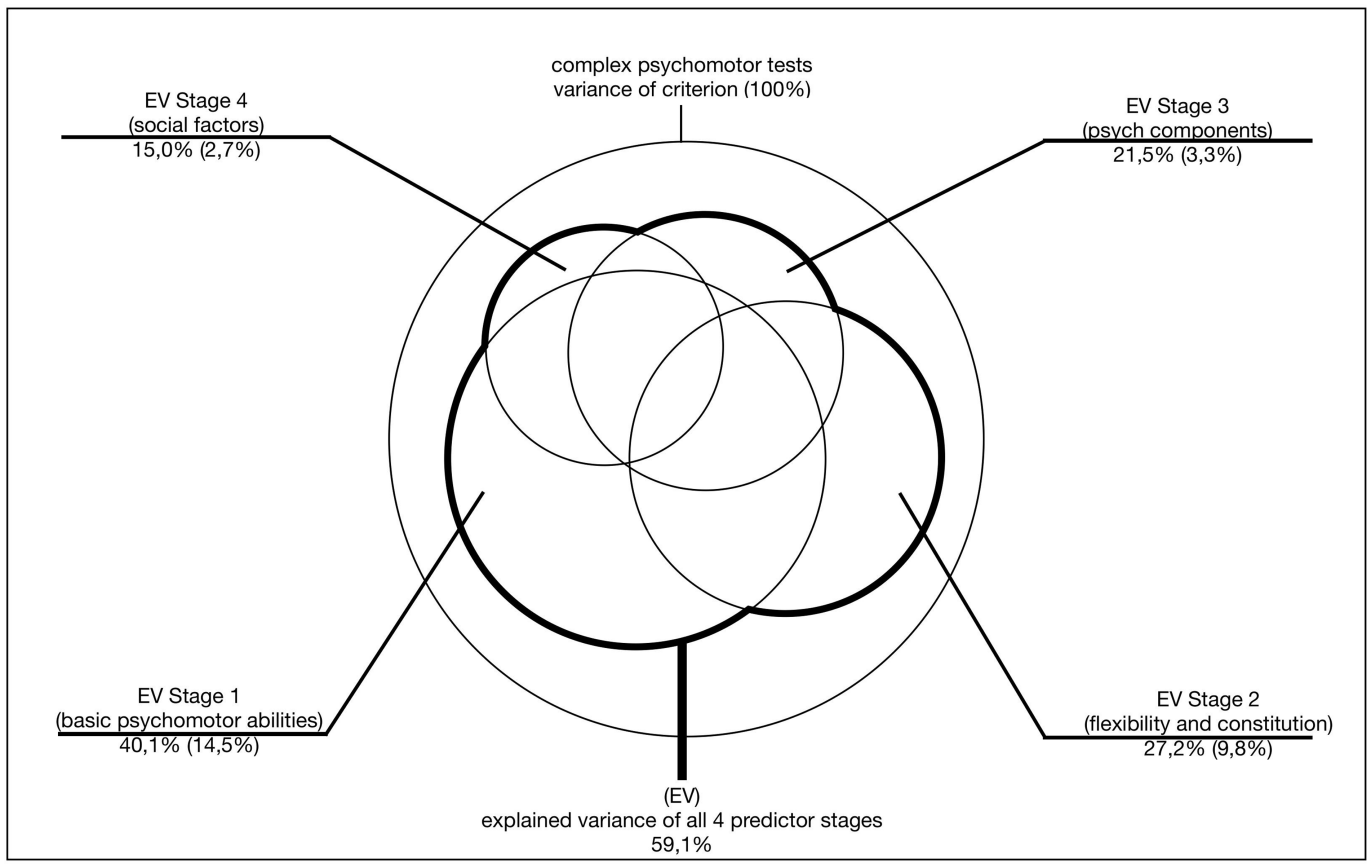
Stage 4 of model testing (Adapted from Bös & Mechling, 1983, p. 289).

- *Predictor Level 1:* BA (= Predictors are the unidimensional **B**asic psychomotor **A**bilities.
- *Predictor Level 2:* BA plus pET
  (= **p**assive **E**nergy **T**ransfer systems, that means the impact of constitution and flexibility (F)).
- *Predictor Level 3:* BA + pET + IS
  (= higher level of **I**nformation **S**ystems (IS).
- *Predictor Level 4:* BA + pET + IS + SS
  (= the influence of **S**ocial **S**ystems, like movement activity and sport-specific, environmental influences (parents, sports club…)

#### 4.2.1 Excursus: systematization of psychomotor abilities

In the following differentiation of psychomotor abilities (Figure 3), we draw on the older approaches of Gundlach (1968) and Pöhlmann (1977). At the *first level*, psychomotor abilities are differentiated into *conditional (energy-related) and coordinative (information-oriented)* psychomotor *abilities. At the second level*, we distinguish the “basic characteristics” of *endurance (E), strength (ST), speed (S) and coordination (C)* as described in sports science and practice.

These conditional strength and endurance psychomotor abilities can be further differentiated based on the duration and intensity of the exercise (third level). A distinction between *aerobic (E-A)* and *anaerobic (E-anA) endurance* as well as *maximum strength (ST-M), speed strength (ST-S)* and *strength endurance (ST-E)* seems to be both necessary and sufficient for issues in school sport, health sport and basic training in competitive sport.

*Speed (S) is differentiated into into action (S-A) and reaction speed (S-R)*. In its complex, sport-specific manifestation as *action speed (S-A)* is the ability to quickly move the body in response to a stimulus. Speed cannot be clearly classified as either a conditional or a coordinative ability. Fast movements are characterized by an optimal interplay between energetic potential and the quality of sensorimotor regulatory processes.

Coordinative psychomotor abilities (C), understood as information-oriented functional potentials, can be differentiated based on the type of sensory regulation and movement actions, depending on the requirement profile. By combining (neuro-)physiological findings with sports-science approaches, Roth (1982) distinguishes between “*coordinative* psychomotor *abilities for controlling movement precision (C-P)”* and “*coordinative abilities under time pressure (C-T).”* Similarly, Bös and Mechling (1983, p. 191) distinguish between *guided* and *ballistic* movements based on the mode of execution.

*Flexibility (F)* is the ability of muscles and joints to move through their full range of motion easily and without pain. Flexibility cannot be clearly assigned to either the conditional or the coordinative domain. Problems arise mainly from the varying approaches of operationalizing flexibility, ranging from anthropometric-biomechanical perspectives to neurophysiological interpretations, such as in stretching. In this context, an anatomical-muscular operationalization was used as “range of motion of the joints.”

At the third level of categorization, a total of 10 psychomotor abilities are thus identified. Among these, three psychomotor abilities (maximum strength (ST-M), aerobic endurance (E-A), and coordinative psychomotor abilities for controlling movement precision (C-P)) are considered uni-dimensional. A detailed analysis of their dimensional structure is provided by Bös and Mechling (1983) and Bös (1987) (Table 1).

**Table 1:** Studies and samples.

No.	Study	Authors	Year	Sample characteristics (n, age, sex)
1	Baseline study	Bös & Mechling	1983	*n* = 342, 10 years, m
2	Follow-up study 1	Multerer	1991	*n* = 111, 20 years, m
3	Follow-up study 2	Schott	2000	*n* = 33, 30 years, m
4	GZM-baseline study	Woll	1995	*n* = 485, 35–55, m & w
5	GZM-follow-up study 2	Bonadt	2016	*n* = 737, 33–75, m & w
6	GZM-follow-up study 1	Wiemann	2023	*n* = 80, 64–84, m & w

### 4.3 Studies and samples

In the baseline study (1) by Bös and Mechling (1983), a cohort of 342 10-year-old boys was comprehensively examined with regard to their psychomotor, psychological, and emotional abilities as well as socialization variables using standardized tests and questionnaires (Figure 2), with the aim of testing the structural model outlined above (Figure 1). Unfortunately, physical education was not coeducational at that time, and for organizational reasons only boys could be tested.

The same group of males was re-examined in two subsequent longitudinal studies (Studies 2 and 3) by Multerer (1991) and Schott (2000). At the three measurement points, the participants in the longitudinal sample were 10 years old (*N* = 342 in 1976, 4th grade), 20 years old (*N* = 111 in 1986), and 30 years old (*N* = 33 in 1996). Studies 2 and 3 examined the same variables as the baseline study (1).

In another longitudinal study (study 4), a community sample of men and women aged 35 to 55 was examined in 1995 (Woll, 1995). The sample was drawn representatively from the municipality’s population register. Psychomotor abilities such as endurance, strength, coordination, and agility were measured using tests. A licenced physician assessed the participants’ health status and measured parameters of metabolic syndrome (i.e., obesity, high blood pressure, cholesterol, blood sugar). After 18 years, these participants, who were then between 53 and 73 years old, were examined again (Study 5, Bonadt, 2016). Another follow-up examination was conducted 9 years later. At that time, the participants were between 64 and 84 years old (Study 6, Wiemann, 2023). The follow-up studies examined the same motor abilities and medical parameters as the initial examination (Study 4).

## 5 Results for the validation of the ability-orientated approach

### 5.1 Quality of the explanatory model for sports-related physical activity performance

#### 5.1.1 Explanation of sports-motor performance (5 complex psychomotor performance tests (question 1 und 2)

In the first explanatory stage, the three uni-dimensional basic psychomotor abilities (BA = maximum strength [ST-M], aerobic endurance [E-A], and coordination in precision tasks [C-P]) were used as predictors to explain performance in 5 complex psychomotor tests (criterion). At the construct level of psychomotor abilities, these can be assigned to the systems of information-oriented movement control and regulation (coordination), or to the energetic systems responsible for energy-flow regulation (condition). The uni-dimensional nature of these basic psychomotor abilities was confirmed through factor analyses and Rasch models (Bös and Mechling, 1983).

Multivariate communality analyses (Cooley and Lohnes, 1976) were computed for statistical analysis. As explained in 4.2, these analyses enable the calculation of total explained variances and their decomposition (Figure 4).

The three basic motor skills explain a total of 40.1% of the variance in complex motor tests. The largest proportion of variance is accounted for by coordination in precision tasks (C-P) (20.6%), followed by aerobic endurance (E-A) (16.9%) and maximum strength (ST-M) (15%). The communality analysis also allows the variance shares to be broken down into their components (Figure 4). Of significant interest are the pure proportions attributable to the individual basic psychomotor abilities. In isolation, coordination in precision tasks explains 10.8% of the variance, maximum strength 10.1% and aerobic endurance 8.5%. Together, this amounts to 29.5%, with the remaining 10.6% accounted for by combined components.

Stage 2 of the model test (BA + pET)

In the second explanatory stage, flexibility and constitution were used as active transmission systems, serving as predictors.

Stages 3 and 4 of the model tests

In two further explanatory stages, psychological components (higher level of information systems, IS) such as intelligence (assessed through CFT2, Brickenkamp, 1997), concentration (assessed through d2 test) and achievement motivation (assessed through LM grid, Brickenkamp, 1997) were measured using validated standard tests (stage 3) and finally, general and sport-specific socialization factors (social systems, SS) were recorded using questionnaires for students and parents (stage 4), and taken into account for the performance explanation.

In the model, these components are referred to as information processing and energy flow control systems, as well as general and sport-specific environmental conditions.

The four predictor stages explained in 4.2 accounted for 59.1% of the variance in complex sport-motor performance, with a multivariate correlation of 0.77. The majority of the explained variance was attributed to the three basic motor dimensions (maximum strength, aerobic endurance, coordination in precision tasks, 40.1%) (stage 1 of the model test).

This 40.1% variance explained in complex psychomotor test performance by uni-dimensional basic psychomotor abilities serves as confirmation of the validity of the chosen dimensional analytical approach.

The total variance explained by the remaining three other predictor blocks ranged from 27.2% (flexibility and constitution, pET) to 21.5% (psychological components, IS) and 15.0% (sport-specific socialization factors, SS).

The “pure” variance explained, taking into account the previous predictor stages, were 2.7% (soc. factors), 3.3% (psych. components) and 9.8% (flexibility and constitution) (Figure 5).

The overall model thus accounts for slightly less than 60% of the variance, providing strong support for the validity of the theoretical approach.

In the original study (Bös and Mechling, 1983, pp. 300ff), cross-validation was also performed. For this purpose, the sample was divided, the analyses were calculated separately and the regression equation weights from one subsample were applied to the other. This procedure demonstrated a high degree of stability in the model testing. The criterion estimate correlations are only approximately 0.1 lower than the multiple correlation coefficients.

#### 5.1.2 Explanation of sporting achievements (question 1 and 2)

The criteria used included the results of the athletics triathlon at the National Youth Games (“Bundesjugendspiele”) and the jumping score.

The four predictor stages show a strong correlation with the National Youth Games (“Bundesjugendspiele”) performance, with a total correlation coefficient of 0.94 (*p* < 0.05), corresponding to an explained variance of 87%. The most significant predictors are the uni-dimensional basic dimensions of aerobic endurance (*r* = 0.47), coordination in precision tasks (*r* = 0.39), and maximum strength (*r* = 0.35).

As the grading scale was only 1–3 for 99.4% of the children, a discriminant analysis was performed to differentiate between the 1–3 grading groups. The two discriminant functions correlated with the predictor set at 0.97 and 0.67, respectively. Once again, the most significant predictors were the previously mentioned basic psychomotor dimensions. In a reclassification, 62% of the children could be placed in the correct grade group based on these predictors.

### 5.2 Stability of psychomotor abilities (question 3)

The follow-up studies referenced above (see Section 4.3) were utilized to examine the long-term generalizability and stability of psychomotor abilities over extended periods.

The results (Table 2) demonstrate a high level of stability in psychomotor abilities over time. For all psychomotor abilities analyzed across different measurement points, the average stability is 0.57.

**Table 2:** Stability of psychomotor abilities.

Psychomotor abilities	Years of comparison/Age *N*				Mean correlation
	1976–86 10–20 (*N* = 111)	76–96 10–30 (*N* = 33)	86–96 20–30 (*N* = 33)	1992–2021 45–75 (*N* = 89)	
Aerobic endurance	0.34	0.14	0.47	0.65	0.40
Maximal strength	0.61	0.47	0.73	0.80	0.65
Coord. in precision tasks	0.48	0.49	0.54	0.57	0.52
Flexibility	0.66	0.67	0.73	0.74	0.70

Among the individual psychomotor abilities, the basic dimensions of maximum strength (*r* = 0.65) and agility (*r* = 0.70) demonstrated the greatest stability.

This stability appears to be consolidated in young adulthood (ages 20 to 30; *r* = 0.62) and even more so in older adulthood (*r* = 0.69). In contrast, stability is less pronounced during childhood and adolescence (ages 10–20; *r* = 0.52), and particularly during the transition from childhood to adulthood (ages 10 to 30; *r* = 0.44).

The basic dimension of aerobic endurance exhibits the lowest average stability coefficient during the 10–30-year period (*r* = 0.14). This can likely be attributed to two main factors: first, maximum oxygen uptake, used as a measure of endurance, is estimated from other endurance tests, and second, physical activity levels in this age group are highly variable. During the dynamic developmental phase of childhood and adolescence, there is greater variability in psychomotor abilities. In contrast, the older sample (ages 45–75) shows the highest stability at age 29 (average *r* = 0.69). In this group, maximum strength is the most stable (*r* = 0.80), followed by flexibility (*r* = 0.74) and aerobic endurance (*r* = 0.65).

Overall, the results presented indicate a high level of stability in psychomotor abilities across the lifespan, underscoring the generalizability of these empirical findings.

The empirically validated dimensions of psychomotor abilities are therefore recommended as core components of curricula for physical education, as well as sports and therapeutic training (Hummel, 2022). Focusing on and emphasizing these dimensions has been shown to yield long-term benefits. This emphasis is likely to have a significant impact on predicting active leisure participation, health development, therapeutic success, and overall lifestyle.

### 5.3 Relevance of psychomotor abilities for health (question 4)

To assess the relevance of the identified psychomotor abilities for health parameters, studies have been conducted using the samples described in Chapter 4.3.

500 men and women were examined every 5 years over a 29-year period, from 1992 to 2021.

At study baseline, participants were aged between 35 and 55 years. To mitigate sample attrition, approximately 100 men and women, each aged 35, were added to the study at every measurement point.

Associations between psychomotor abilities and health parameters were examined at two measurement points, in 2015 and 2021, using a distinct methodological approach. The health parameter analyzed was the presence of metabolic syndrome (METS), which is defined by the co-occurrence of obesity (abdominal circumference >102 cm for men or >88 cm for women), insulin resistance (blood glucose >100 mg/dL), elevated cholesterol levels (triglycerides >50 mg/dL), and high blood pressure (systolic >130 mmHg, diastolic >85 mmHg).

In his work, Bonadt (2016, pp. 175ff) analyzed the impact of fitness level on the development of metabolic syndrome. He focused on two of the identified basic motor dimensions (“aerobic endurance” and “strength”), investigating whether a high level of these psychomotor abilities is associated with lower occurrence of metabolic syndrome.

In his analysis, there were 914 “person-years at risk” (Dos Santos Silva, 1999) among individuals who were consistently fit, compared to 4,037 among unfit peers. Metabolic syndrome occurred 6 times in the fit group and 74 times in the unfit group, resulting in a rate ratio of 3.6, which is statistically significant (*p* < 0.01). “Person Years at Risk” refers to the total of all time periods across individuals, with the assumption that metabolic syndrome (METS) has not yet occurred up to the point it does occur (Bonadt, 2016, p. 115).

A detailed analysis of the dimensions of strength and endurance reveals similar results. Fitter individuals have significantly fewer “person-years at risk” compared to those who are less fit, and are also significantly less likely to develop metabolic syndrome (Bonadt, 2016, p. 176).

These results indicate that fit individuals have a significantly lower risk of developing metabolic syndrome compared to those who are unfit, with no significant gender differences observed.

Wiemann (2023) adopted a slightly different approach in his study, focusing on whether motor performance measured in early 1992 was associated with the development of metabolic syndrome by 2021. He included 89 participants (48 men) in the analysis and found a significant correlation of *r* = 0.30 (*p* < 0.01) for the overall “motor performance score” after 29 years. No sex differences were observed. While the correlations for individual psychomotor abilities were not significant, the author interprets this finding over such a long period as suggesting that early-life performance may serve as a meaningful predictor of later disease outcomes.

In a cross-sectional analysis conducted by Woll and Bös (1994) on the initial sample from 1992 (*N* = 500, *m* = 250, ages 35–55), motor performance showed a correlation of 0.39 (p. 71) with the physician’s assessment of health. This corresponds to an explained variance of 15% and further supports the role of fitness in health outcomes.

In conclusion, these results confirm the relevance of the ability approach and, by extension, the theoretical health models that posit a relationship between psychomotor performance and health, as seen in both the risk-factor and the salutogenesis model (Cleven et al., 2022). A distinctive aspect of the present findings is that these relationships are not only evident in cross-sectional analyses but also persist in longitudinal analyses.

## 6 Discussion

### 6.1 Limits of ability-orientated approaches

Based on a theoretically described structural model for explaining physical and sports-related movement performance, the underlying psychomotor abilities were first examined for uni-dimensionality. The explanatory power of these uni-dimensional basic dimensions in accounting for complex movement performance on the basis of the performance model was confirmed. As a result, these psychomotor abilities were validated as influential and effective factors in practical application. In socially significant domains such as health and fitness, physiotherapy, or sport-talent scouting, useful findings for targeted intervention can be expected. Whether these results are transferable to other areas of training and movement as physiotherapy, sport-talent scouting or monitoring of public health remains an open question for further discussion.

In general, psychomotor abilities, understood as behavioral dispositions, only manifest under specific situational conditions. As a result, the predictive power of individual psychomotor abilities for motor behavior is often reduced. This is because both the isolated situation and the interaction between individual psychomotor abilities and situational factors must be considered as explanations for specific behaviors. This partitioning of variance applies regardless of whether the relationship between personal characteristics and situational conditions is examined experimentally or through correlational study designs. While experimental approaches often allow for more precise definitions of both condition and criterion variables, enabling better control of error variance, the field of sports science has now shifted in favor of experimental approaches. These approaches tend to yield more accurate predictions than correlational studies, particularly when addressing peak performance or isolated cognitive and motor skills. For instance, it has been demonstrated that strong prior *knowledge of principles*, *rules*, and *laws* exerts a greater influence on motor performance than “general psychomotor abilities” (Weinert et al., 1991). This raises the question of which research methodological strategies, experimental or correlational-ability-based, can provide better indications for methodological-didactic measures in practice for an application- and practice-oriented approach. However, prior to the knowledge of the principles, rules and laws of motor performance realization requires time, and extensive experience is needed to produce better performance predictions, especially in high-performance contexts. The ability approach offers a valuable framework for designing such developmental pathways in basic abilities.

### 6.2 Scope of the ability-orientated approach in psychology and sports science—where to position?

Sports-science motor behavior research in the narrower sense of “motor learning, motor control, neuro- and sensorimotor,” but also “metabolic and biogenetic,” has long adhered to the credo of experimental psychology in its pursuit of underlying mechanisms. In terms of content, there has been a strong orientation towards “motor neuroscience” or “computational neuroscience” in recent years (Franklin and Wolpert, 2011).

### 6.3 Critical discussion of psychomotor abilities in sports science

The ability-based approach to explaining physical-motor performance has often been criticized on basic scientific and causal-analytical grounds. This is particularly true for coordinative psychomotor abilities (Hossner and Künzell, 2022, pp. 354ff). While conditional psychomotor abilities are generally accepted, due to their demonstrated stability and biological basis in systems such as musculature and the cardiovascular system, the classification of coordinative psychomotor abilities as “psychomotor abilities” has been increasingly questioned in light of recent perceptual-motor research. This skepticism is largely rooted in previous research results by Hossner.

Building on foundational scientific theories, Hossner’s modular approach, drawing on Fodor’s (1983) “modularity of mind” framework from cognitive psychology, represents an attempt to combine practical and basic knowledge for sports science. According to Hossner, these are explanatory approaches to the problem of movement coordination as an ability. It is postulated that the modular approach offers positive prospects both for sports practice, particularly in terms of the consistent structuring of technique training, and for basic motor-behavior research. Although the concept of modularity currently functions primarily as a metaphor, it is considered as of substantial heuristic value (Hossner, 1995, p. 233). In this context, a motor module “is defined as an informationally encapsulated component of the movement control system that facilitates structural learning and thus enables cross-task transfer effects” (Hossner and Künzell, 2022, p. 377). Key performance factors such as movement duration, speed, accuracy, and temporal structure are incorporated. The aim is to create a modular system for technique training from identified modular technique components. According to Hossner, coordination should be regarded less as a prerequisite than is the case of conditional psychomotor abilities.

From our ability-based perspective, the following should be noted here: If modules initially have *a heuristic value* and are understood as an “*informationally encapsulated part of the movement control systems*,” the question of module content arises. Even with psychomotor abilities as constructs, there is no requirement that they must be represented by an organ. Endurance and strength as constructs are also characterized by the way they function in detail, the respective metabolism, without the need to consider the detailed processes (such as muscle contraction.) for the occurrence of a movement. The same could be said of the central nervous system. The structures and processes that control movement are being described more and more precisely by molecular biology and radiology (MRI). But even in detail, it is sometimes necessary to resort to “constructs” as temporary aids as is the case in robotics. The central nervous system is also trained and changes, for example, through enriched environmental design or by varying movement sequences. In our understanding, this variety of procedural changes and new network connections can be more easily summarized in larger units such as “coordinative psychomotor abilities,” which form a perceptual-motor whole. The externally visible movements as specific coordination performances can then be diagnosed by means of perceptual-motor behavioral tests. A current explanatory approach that is consistent with this understanding emphasizes the emergence of specific “perceptuomotor function modules” that become established neurobiologically through plasticity based on the specific information content of certain perceptual references through recurring training or action situations: For example, in visual perception, the use of components of optic flow (Gibson, 1979) to specify one’s own running direction and speed, or the rate of expansion and symmetry of the background overlap of an approaching ball to specify the catch location and time (Effenberg and Schmitz, 2022). Repeated similar actions during practice and training enable the perceptual system to detect task- or action-specific central information components, often in the form of invariant structural components in the perceptual spectrum of the subject-environment constellation specified by the individual’s motor behavior.

## 7 Conclusion and outlook

Researchers with a sports-science or psychomotor orientation have developed ability-oriented concepts and models that enable a multidimensional description and assessment of motor functions. These frameworks provide a solid basis for reliably predicting psychomotor performance behavior. The empirical analyses and findings presented in this article support the effectiveness of such approaches. It is well established that such psychomotor abilities demonstrate a high degree of stability across extended periods of the lifespan. Particular emphasis is placed on the one-dimensional components “*aerobic endurance*, *maximum strength* and *coordination in precision tasks*,” referred to here as basic motor dimensions.

The distinctions between motor dimensions outlined here are recommended for use in individual psychological diagnosis and assessment. However, it must be acknowledged that situational factors, and their interaction with individual psychomotor abilities, play a significant role in motor behavior and may limit the explanatory and predictive power of psychomotor abilities. In the long term, it remains uncertain how computational, neuromechanical, or mathematical modeling approaches to motor control processes might contribute to more precise descriptions, dimensional frameworks, and deeper analyses of human abilities. These research directions constitute highly valuable and promising directions for future research. Concurrently, fundamental research in the fields of sensorimotor control and motor learning is progressing rapidly. It remains an open question to what extent these advancements will enrich the concepts of psychomotor abilities and modules, or potentially render them obsolete.

Complex approaches and models for describing and explaining physical and athletic performance, such as the Grand Unified Theory of Sports Performance (GUT), the concept of agility (Nicolescu, 2002; Nimphius et al., 2017), and the ability approach, are grounded in differing theoretical assumptions and vary in their levels of analytical differentiation. Despite these differences, they share a common objective: to enhance the understanding of physical and athletic psychomotor performance by employing appropriate analytical units and methodologies. On the one hand, the aim is to gain a better understanding of the fundamental mechanisms and their interactions in order to advance the development of theory. On the other hand, the aim is to identify behavioral interventions to improve and stabilize psychomotor performance. The criterion for all of them is the construct of *psychomotor performance*.

**Figure 6:**
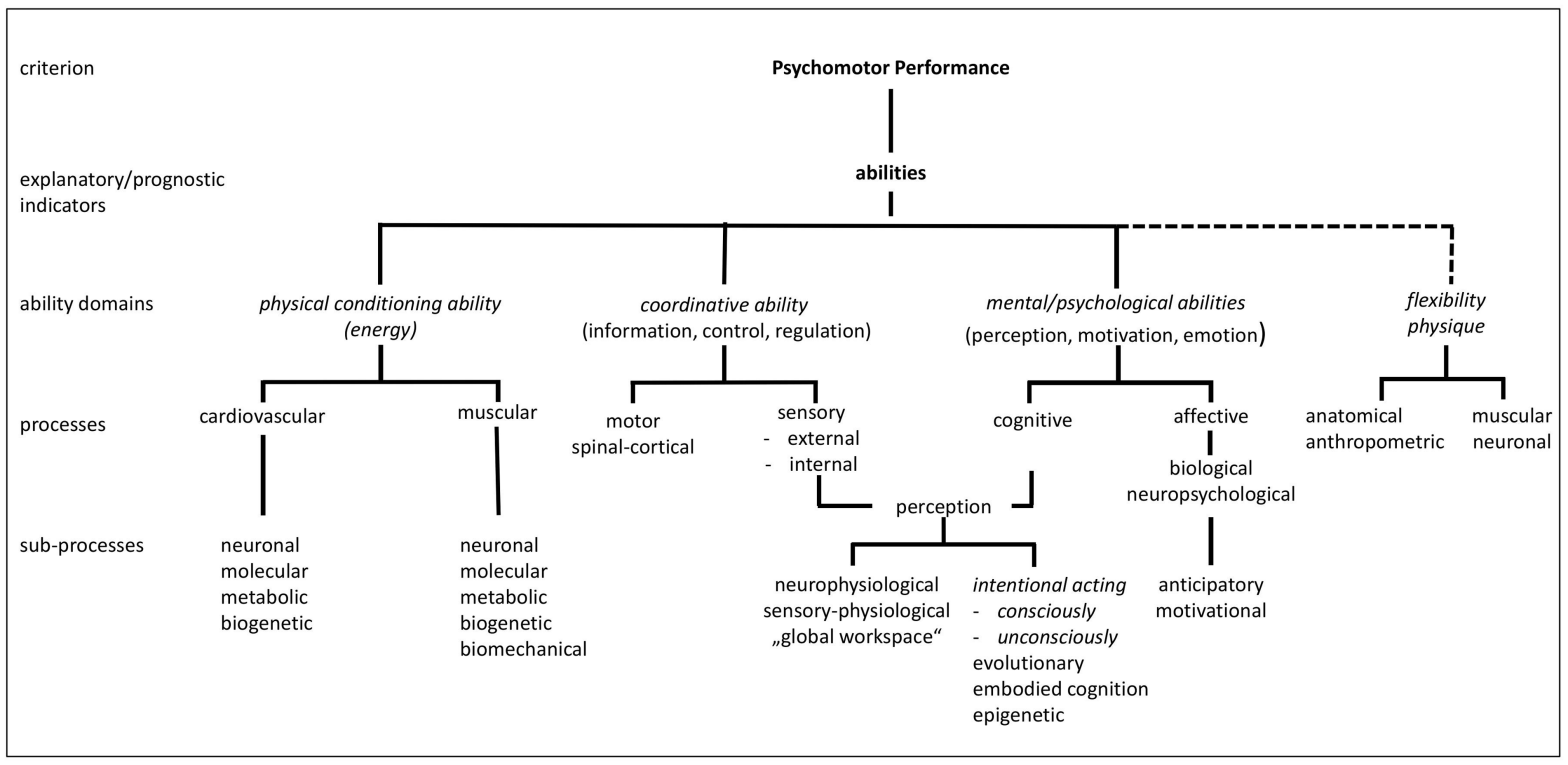
Perspectives on the interdisciplinary approach to psychomotor performance (Adapted from Mechling, 2021, p. 139f).

Interdisciplinary research is regarded as essential and is actively promoted across all theoretical approaches. The model presented in Figure 6 (Mechling, 2021, p. 243) serves as a proposal that illustrates the interconnections among these approaches. It facilitates the alignment and integration of diverse perspectives, highlighting their respective contributions and thereby fostering interdisciplinary dialogue. This model also opens the door to potential transdisciplinary collaboration (Mittelstraß, 2002; Nicolescu, 2002).

*Psychomotor performance* (level 1) serves as the criterion in this model. Psychomotor abilities (level 2) are treated as explanatory and prognostic factors, further differentiated into distinct areas of ability (level 3) (Mechling, 2021, p. 243). At the levels of processes (level 4) and sub-processes (level 5), the structures involved, as well as their functional and procedural bases, are only briefly outlined. A further possible differentiation below the level of “sub-processes” is not discussed here. Similarly, the interface between the ability areas (level 3) and the underlying processes and sub-processes (levels 4 and 5) remains unexplored in this context. These processes are rooted in biogenetic and molecular mechanisms that can be analyzed down to substrates and specific, measurable parameters. This approach aligns with the idea that our current understanding of the integration within and between these processes is based on hypothetical constructs, which can be refined over time. However, it is important to acknowledge that the more detailed the analysis becomes, the more challenging it is to draw meaningful conclusions about the behavioral level of physical performance (level 1) and, consequently, to intervene in the complex reality of human activities, such as in sports, physical education, or health-related exercise.

Age-related changes across and between the different levels have yet to be thoroughly investigated. The levels illustrated in Figure 6 including the ability domains and the process-related mechanisms described, are based on the assumption that they collectively contribute to physical performance and, by extension, to overall behavior.

The descriptive categories at each level can be influenced by development and intervention. The assumption of reciprocity between the levels and their units underscores the importance of specific interdisciplinary research as well as an understanding of how to integrate its findings into practical contexts such as educational and therapeutic interventions.

The concept of successive validation of components, such as domains of ability, draws on Neumann’s contribution to attention theories (“from metaphors to mechanisms,” Neumann, 1992, p. 92). Data from diverse disciplines (e.g., neurophysiology, neuropsychology, molecular biology) and collected through various methodologies (e.g., psychological experiments, individual inferences) could be integrated by “vertical integration” using the same putative mechanism. “Where there are similarities, it can be assumed that the component in question does indeed exist as an ability” (p. 92). Such components can then be validated in both application-oriented and basic research-oriented ways.

### 7.1 Limitations of the research

The primary limitation of this paper is that the theoretical model presented could not be fully operationalized and tested, due to the complexity of explanatory models for sports-related movement performance. It is important to note that, in practical research, the effort required to assess complex units of analysis using ability and behavioral tests is far greater than simply relying on individual biomedical parameters.

Another limitation is that the original study was conducted exclusively with boys. At the time, physical education was not co-educational, making the inclusion of a sample of girls beyond the scope of the study.

In the follow-up studies on the stability of psychomotor abilities, the study sample progressively decreased in size. This reduction may introduce selection biases that could influence the results. However, it is likely that selection reduces variance, which may lead to higher stability in unselected samples. Selection effects also apply to the adult sample when examining the predictive power of psychomotor abilities for health.

### 7.2 Strengths of the research

An effort has been made to formulate a comprehensive model for explaining sports-related movement performance and to make it amenable to empirical testing. Despite the aforementioned weaknesses, this endeavor holds significant importance in sports-science and behavioral movement research. The statistical methods employed, such as the Rasch model and communalities analysis, are rarely seen in other approaches. Over nearly 40 years, this performance-oriented approach has been embraced and empirically tested in several follow-up studies. The long-term findings have attracted considerable attention, with numerous partial results already published in high-impact journals (Tittlbach et al., 2017; Wiemann et al., 2024). However, a complex review like the one presented in this article has yet to be published.

## Data Availability

The datasets used in this study are available on request. Requests to access these datasets should be directed to Klaus Bös, boes@kit.edu.
